# The effect of probiotics on ıntestinal motility in an experimental short bowel model 1

**DOI:** 10.1590/s0102-865020200080000004

**Published:** 2020-09-07

**Authors:** Mehmet Metin, Ahmet Altun, Gökhan Köylüoğlu

**Affiliations:** I MD, Department of Pediatric Surgery , Faculty of Medicine , Cumhuriyet University , Sivas , and Department of Pediatric Surgery , Hitit University Erol Olçok Training and Research Hospital , Corum , Turkey . Conception and design of the study, acquisition of data, statistical analysis, manuscript writing, critical revision, final approval.; Department of Pediatric Surgery, Hitit University Erol Olçok Training and Research Hospital, Corum, Turkey; II MD, Department of Pharmacology , Faculty of Medicine , Cumhuriyet University , Sivas , Turkey . Conception and design of the study, acquisition of data, statistical analysis, manuscript writing.; III MD, Department of Pediatric Surgery , Faculty of Medicine , Cumhuriyet University , Sivas , Turkey . Critical revision, final approval.

**Keywords:** Short Bowel Syndrome, Gastrointestinal Motility, Probiotics, Amplitude, Rats

## Abstract

**Purpose:**

To investigate the effect of probiotics on spontaneous contractions of smooth muscle isolated from jejunum and ileum of rat model.

**Methods:**

Four rat groups were created (n=8, in each) including control (Group 1), control+probiotic (Group 2), short bowel (Group 3), and short bowel+probiotic (Group 4). Groups 1 and 2 underwent sham operation, Groups 3 and 4 underwent massive bowel resection. Bifidobacterium Lactis was administered in Groups 2 and 4 daily (P.O.) for three weeks. On postoperative week 3, rats were sacrificed, and jejunum and ileum smooth muscle were isolated for organ bath. Muscle contraction changes were analyzed before and after addition of antagonists.

**Results:**

Short bowel group exhibited increased amplitude and frequency of spontaneous contractions. The addition of probiotics significantly decreased enhanced amplitude and frequency of bowel contraction in short bowel group and returned to control values. L-NNA increased amplitude and frequency of contractions in all groups. While indomethacin and nimesulide increased the amplitude in all groups, the frequency was only increased in jejunum. Hexamethonium and tetrodotoxin did not change the contraction characteristics in all groups.

**Conclusion:**

We suggest that early use of probiotics may significantly regulate bowel motility, and accordingly improve absorption of nutrients in short bowel syndrome.

## Introduction

% In recent years, numerous publications have shown that probiotic bacteria are highly beneficial for human health ^1 - 6^ . It has been demonstrated that they regulate flora by colonizing in the intestinal flora, prevent the colonization of pathogenic bacteria in the bowels ^7^ , enhance immune resistance ^8 - 13^ , prevent diarrhea and constipation, are beneficial in cancerous and inflammatory bowel diseases, regulate motility and stool frequency, and experimentally they reduce bacterial translocation ^14 - 21^ . However, the effects of which probiotics regulate motility are unknown.

Short Bowel Syndrome (SBS), is a clinical condition that develops as a result of reduced bowel transit time due to the removal of a significant portion of the small intestines or compromised absorptive functions ^22^ . The follow-up and treatment of SBS are somewhat tricky, and the survival of patients is closely associated with the adaptation capacity of the remaining large bowel ^23^ . Until now, many experimental studies that used different substances to improve bowel adaptation in SBS ^24^ have been conducted. Although many SBS studies defined luminal nutrition (long chain fatty acids, sugar and glutamine) ^25 , 26^ , pancreaticobiliary secretion ^27^ , and hormones and mediators (growth hormone, enteroglucagon, neurotensin, insulin-like growth factor-1, epidermal growth factor etc.) ^28^ as examples of factors that improve bowel adaptation, very few of the elements with proven efficacy are used clinically ^24^ . Although a lot of clinical research has been conducted to understand the underlying physiopathology of SBS, the number of studies that examine the effects of the disease on the bowels in vitro is little if any ^29^ . With this goal, we created an animal model of short bowel syndrome in rats and examined the functional changes that occurred over a three-week period. We formed another group that was fed with probiotics that are claimed to have beneficial clinical effects, and we evaluated the changes caused by the probiotics. We performed all the evaluations both in the jejunum and in the ileum. Besides this, we tried to explain the reasons underlying the changes in the presence of antagonists which aimed to target different pathways participating in the smooth muscle contraction.

## Methods

This study was conducted in the Experimental Animals Research, and the Pharmacology Laboratory of Faculty of Medicine in Cumhuriyet University, after approval was obtained from the Cumhuriyet University Medical Faculty Ethical Board (Date: 03.05.2007 No: 100). The experimental study was financially supported by the Scientific Research Projects Commission Directorship of the Cumhuriyet University (Project number: T-326).

### Animals

Albino male rats were used as test subjects. The short bowel model utilized in this study was applied by Eizaguirre *et al* . ^16^ as defined before. The study was conducted with 32 adult Winstar rats weighing 200-400 g. Rats were taken care of under standard laboratory conditions. Animals were divided into 4 experimental groups including control (Group 1, n=8), control + probiotic (Group 2, n=8), short bowel (Group 3, n=8), short bowel + probiotic (Group 4, n=8).

### Surgical procedure

Animals were administrated 3 mg/kg of xylazine hydrochloride (Rompun, 50 cc vial, Bayer) + 90 mg/kg of ketamine hydrochloride (Ketasol 10%10 cc vial, Richterpharma ag) intramuscularly to obtain anesthesia. After the operation, 4 mg/kg of Carprofen (Rimadyl 20 cc vial, Pfizer) was delivered subcutaneously as an analgesic. After anesthesia had been achieved, the abdomen was cleansed using povidone-iodine (Betadine 10%) with the rat placed in supine position. A 4 cm incision was made to open the abdomen. In the control and control+probiotics groups (Groups 1 and 2), rats underwent a sham operation. The bowel was cut so that it would correspond to the middle point between the treitz ligament and the cecum; a single layer anastomosis was performed using 6.0 silk sutures (Ethicon). In the short bowel and short bowel+probiotics groups (Groups 3 and 4), the intestines between 10 cm distal from the treitz ligament and 10 cm proximal to the ileocecal area were resected, and a single layer anastomosis was performed. After these procedures, the abdomen was closed anatomically using 3/0 silk (Ethicon) sutures. One day after the operation, 7.8 x 10 8 of the probiotic (Bifidobacterium lactis) prepared in the Microbiology Laboratory of the Cumhuriyet University was given once daily via nasogastric gavage to the control+probiotics and short bowel and probiotics groups (Groups 2 and 4). Three weeks later all subjects in the control group (Group 1) and the short bowel group (Group 3) were sacrificed by delivering 200 mg/kg of thiopental sodium (pental sodium 1 gr, I.E. Ulagay Ilac Sanayi). A median incision was made to open the abdomen, 3 cm long ileal and jejunal tissue samples were taken 1 cm proximal from and 1 cm distal to the anastomosis line.

### The preparation of the tissue samples

The tissue sample obtained was immediately placed in a Krebs-Bicarbonate solution that was aerated using 95% oxygen (O _2_ ) and 5% carbon dioxide (CO _2_ ). The components added are 120 mM/L sodium chloride (NaCl), 4.6 mM/L potassium chloride (KCl), 2.5 mM/L calcium chloride (CaCl _2_ ), 1.2 mM/L magnesium chloride (MgCl _2_ ), 22 mM/L sodium bicarbonate (NaHCO _3_ ), 1.14 mM/L sodium dihydrogen phosphate (NaH _2_ PO _4_ ) and 11.5 mM/L glucose. After tissue samples had been brought to the pharmacology lab, whole layer segments were taken separately from the ileum and jejunum in all groups. They were placed into the tissue bath freshly prepared with an aerated Krebs-Bicarbonate solution at 37 ^o^ C from the direction of circular muscles. Both ends were attached to the isometric transducer (Grass FT 03, Quincy, MA, USA) in a 10 mL tissue bath and 1-1.5 g load was loaded. It was waited thirty minutes for stable basal tissue contraction curves to be obtained.

### Isometric measurements

#### KCl contraction responses

The rats’ ileal and jejunal tissues obtained from the control and experiment groups were treated in an 80 mM KCl tissue bath to test the contraction strength of the tissue before any medications were delivered. The contraction responses are presented as percentage ( Table 1 ).

**Table 1:** The jejunum and ileum KCl values in grams. The findings were presented as the mean ± standard deviation of 8 subjects.

	Control	Short intestine	Pro + control	Pro + Short intestine	*P*
Ileum	2.54 ± 0.20	2.80 ± 0.24	2.38 ± 0.27	2.72 ± 0.24	0.095
Jejunum	2.05 ± 0.26	1.96 ± 0.24	1.90 ± 0.22	2.00 ± 0.25	0.467

#### Amplitude and frequency responses

After the KCl responses, the amplitudes of the spontaneous contractions of the ileal and jejunal tissues of the control, control + probiotic, short bowel, and short bowel + probiotic groups were recorded. They were calculated as the percentages of the responses in 80 mM KCl and graphics were drawn ( Table 2 ). Afterwards, the nitric oxide synthase (NOS) inhibitor NG-nitro-L-arginine (L-NNA, 10-5M), the cyclooxygenase-2 (COX-2) inhibitor indomethacin (10-6M), the selective COX inhibitor nimesulide (10-6 M), the ganglion blocker hexamethonium (10-4 M) and the Na channel blocker tetrodotoxin (10 -7 M) were added to the medium and the muscle segments were rested for 30 minutes. A polygraph (Grass Model 79 E-USA) was used to record isometric contractions. All experiments were performed separately for each one of the four groups.

**Table 2:** The comparative mean contraction values of ileal and jejunal smooth muscles in 80 mM KCl obtained in rats from all groups in percentages. The findings were presented as the mean ± standard deviation of 8 subjects.

	Control	Short intestine	Pro + control	Pro + Short intestine	*P values*	Post-hoc *P* values
Ileum	80.6 ± 6.8	120.6 ± 7.0*	72.2 ± 6.0	96.8 ± 7.6	<0.001*	1-2; <0.001* 1-3; 0.06 1-4; 0.053
Jejunum	60.7 ± 4.5	80.8 ± 5.0*	55.2 ± 4.7	66.2 ± 4.3	0.001*	1-2; <0.001* 1-3; 0.093 1-4; 0.075

* *P* < 0.01 Its difference from the control group is significant.

The amplitudes of the smooth muscle spontaneous contractions of ileal and jejunal tissues obtained from the experiment and control groups were compared to the KCl response with regard to the presence or lack of antagonist agents in the environment and were calculated as a percentage. The frequency responses have been computed as number/minute over 10 minutes depending on whether antagonist medications are present in the medium.

### Medications

The chemical medications L-NNA, indomethacin, nimesulide, hexamethonium and tetrodotoxin used in this study were acquired from Sigma-Aldrich (St. Louis, MO, USA). All chemical medications were dissolved in water except tetrodotoxin. Tetrodotoxin was dissolved in a citrate solution [(50 mM citric acid and 48 mM sodium dihydrogen phosphate (NaH _2_ PO _4_ )]. The chemicals used in the experiments were prepared daily. Each antagonist medication was delivered to the groups at the same amount and interval.

### Statistical analysis

The statistical analyses were performed using SPSS (Version 22.0, SPSS INC., Chicago, IL, USA). The assumptions of normal distribution were evaluated using the Kolmogorov-Smirnov and Shapiro-Wilk. The homogeneity of the variations was assessed using the Levene test. All continuous data are presented as a mean ± standard deviation. In order to compare more than two independent groups, the ANOVA (Variance Analysis) test was used in cases where parametric assumptions were met, and the non-parametric Kruskal-Wallis variance analysis test was used in cases where assumptions were not met. To identify the group that caused the difference, the Post-hoc test the Bonferroni test was used to compare two variables after the variance analysis. Statistical significance was set at *P* < 0.05.

## Results

The results of our study, which investigated the changes in bowel motility and the effects of probiotic treatment on an experimental short bowel model we created in rats, are presented below.

**Figure 1:**
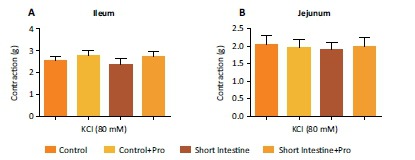
The mean contraction values obtained in 80 mM KCl in the smooth muscle of ileum ( A ) and jejunum ( B ) in rats from all groups.

No statistically significant difference was detected between the contraction responses of the ileal and jejunal smooth muscles of the control, short bowel, control+probiotic, and short bowel+probiotic groups in 80 mM KCl ( *P* = 0.095, *P* = 0.467, respectively) ( Table 1 ) ( Fig. 1 ).

**Figure 2:**
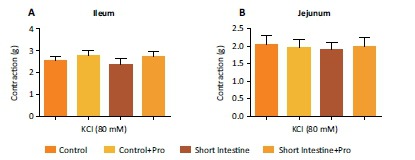
The comparative mean contraction values of the A -ileum and B -jejunum smooth muscles in 80 mM KCl obtained from rats in the control, short intestine, pro + control, and pro + short intestine groups. The findings were presented as the mean ± standard deviation of 8 experiments. * *P* <0.05 Its difference from the control group is significant.

In the short bowel group (Group 3), the amplitude responses in both ileal and jejunal tissues ( *P* <0.001, *P* =0.001, respectively) were significantly higher than the responses of the control (Group 1), the control + probiotic (Group 2), and the short bowel + probiotic (Group 4) groups. No statistically significant difference was observed between control, control+probiotic, and short bowel+probiotic groups ( Table 2 ) (Fig. 2). Likewise, the frequency responses ( *P* <0.001, *P* =0.01, respectively) in the jejunal and ileal tissues of the short bowel group (Group 2) were increased in comparison to the all other groups (Group 1, Group 3, and Group 4). No statistically significant difference was observed between control (Group 1), the control + probiotic (Group 3), and the short bowel + probiotic (Group 4) groups ( Table 3 ) (Fig. 2). The decrease of the contraction responses, for example, the amplitude and frequency to control values, particularly in the short bowel group that was delivered probiotics (short bowel+probiotics, Group 4) shows that probiotics regulate the motility of the short bowel.

**Table 3:** The comparative mean contraction values of ileal and jejunal smooth muscle in 80 mM obtained in rats from the control (grup 1), short intestine (grup 3), pro + control (grup 2), and pro + short intestine (grup 4) groups in percentages (Frequency percentages; number/10 minutes). The findings were presented as the mean ± standard deviation of 8 subjects.

	Control	Short intestine	Pro + control	Pro + Short intestine	*P*	Post-hoc *P* values
Ileum	27.2 ± 4,2	42.8 ± 3.9*	24.0 ± 3.6	32.6 ± 4.1	<0.001*	1-2; <0.001* 1-3; 0.372 1-4; 0.063
Jejunum	20.2 ± 3,0	29.2 ± 3.2*	17.8 ± 3.4	23.8 ± 3.3	0.010*	1-2; <0.001* 1-3; 0.471 1-4;0.117

*P* < 0.05 Its difference from the control group is significant

**Figure 3:**
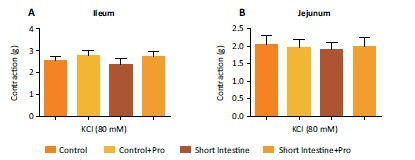
The changes caused by Krebs bicarbonate solution (vehicle), L-NNA, indomethacin, nimesulide, hexamethonium, and tetrodotoxin in the amplitude of spontaneous smooth muscle contractions in the ileum obtained from rats in all groups. A - Control, B - Short intestine, C - Control pro, D - Short intestine - pro. The findings were presented as the mean ± standard deviation of 8 experiments. * *P* < 0.01 Its difference from the control group is significant.

**Figure 4:**
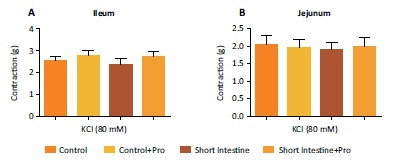
The changes induced by Krebs bicarbonate solution (vehicle), L-NNA, indomethacin, nimesulide, hexamethonium, and tetrodotoxin on the amplitude of spontaneous jejunal smooth muscle contractions obtained in all groups. A - Control, B - Short intestine, C - Control pro, D - Short intestine - pro. The findings were presented as the mean ± standard deviation of 8 experiments. * < 0.001 The difference from the control group is significant.

In order to see the underlying mechanism of the effects, observed at the first part of the study, amplitude and frequency responses were re-evaluated in the present of antagonist agents in both ileum and jejunum. The nitric oxide synthase (NOS) enzyme inhibitor L-NNA (10 ^- 5^ M), the COX inhibitor indomethacin (10 ^- 6^ M), the selective COX-2 inhibitor nimesulide (10 ^- 6^ M), the nicotinic receptor blocker hexamethonium (10 ^- 4^ M), and tetrodotoxin (10 ^- 7^ M) that blocks voltage-gated sodium channels were used. A significant difference was detected across the groups (For the ileum; *P* <0.001, *P* <0.001, *P* =0.002, *P* <0.001, respectively), (For the jejunum; *P* <0.001, *P* <0.001, *P* <0.001, *P* <0.001, respectively). L-NNA, indomethacin, and nimesulide increased the amplitude of the contractions in both jejunal and ileal tissues. This is achieved by inhibiting the nitrergic system and prostaglandin synthesis. Tetrodotoxin and hexamethonium did not alter amplitude levels (Tables 4 and 5) (Figs. 3 and 4). It is believed that probiotics reduce amplitude levels in short bowels by increasing the synthesis of nitric oxide and prostaglandins.

**Table 4:** Amplitude changes in the presence of antagonists in ileal tissue (% KCl). The changes induced by Krebs bicarbonate solution (vehicle), L-NNA, indomethacin, nimesulide, hexamethonium, and tetrodotoxin in the amplitude of spontaneous ileal smooth muscle contractions obtained in rats from all groups are shown in Group 1, Group 2, Group 3, and Group 4. Before the measurement, all tissues were incubated with antagonist agents for 30 minutes. The findings were presented as the mean ± standard deviation of 8 subjects.

Ileum	Control	Short intestine	Pro + control	Pro + Short intestine
Vehicle	80.6 ± 6.6	120.6 ± 7.0	72.2 ± 6.6	96.8 ± 7.6
L-NNA	124.2 ± 8.2*	168.8 ± 8.1*	94.2 ± 7.1*	134.2 ± 7.0*
Indomethacin	112.0 ± 7.2*	156.8 ± 7.2*	90.6 ± 6.4*	128.2 ± 7.2*
Nimesulide	102.8 ± 6.9*	148.2 ± 6.6*	87.2 ± 5.4*	118.4 ± 6.8*
Hexamethonium	70.9 ± 6.8	110.5 ± 7.0	66.6 ± 5.0	89.7 ± 6.4
Tetrodotoxin	72.3 ± 6.5	107.2 ± 6.4	64.2 ± 5.2	82.4 ± 6.3
*P* values	<0.001*	<0.001*	0.002*	<0.001*
Post hoc *P* values	1-2; <0.001*	1-2; <0.001*	1-2; <0.001*	1-2; <0.001*
	1-3; <0.001*	1-3; <0.001*	1-3; <0.001*	1-3; <0.001*
	1-4; <0.001*	1-4; <0.001*	1-4; <0.001*	1-4; <0.001*

*P* < 0.01 Its difference from the control group is significant.

**Table 5:** Amplitude changes in the jejunal tissue in the presence of antagonists (%KCl). Changes induced by Krebs bicarbonate solution (vehicle), L-NNA, indomethacin, nimesulide, hexamethonium, and tetrodotoxin in spontaneous jejunal smooth muscle contractions obtained in rats from all groups are shown in Group 1, Group 2, Group 3, and Group 4. Before the measurement, all tissues were incubated with antagonist agents for 30 minutes. The findings were presented as the mean ± standard deviation of 8 subjects.

Jejunum	Control	Short intestine	Pro + control	Pro + Short intestine
Vehicle	60.7 ± 4.5	80.8 ± 5.0	55.2 ± 4.7	66.2 ± 4.3
L-NNA	88.8 ± 5.2*	113.6 ± 6.1*	77.4 ± 4.5*	100.8 ± 5.2*
Indomethacin	84.2 ± 4.8*	106.3 ± 5.2*	72.2 ± 4.3*	93.4 ± 4.6*
Nimesulide	80.7 ± 4.3*	100.2 ± 4.8*	69.8 ± 4.6*	88.8 ± 4.4*
Hexamethonium	54.1 ± 4.0	73.4 ± 4.3	48.9 ± 4.0	59.0 ± 4.5
Tetrodotoxin	52.6 ± 4.4	70.0 ± 5.2	45.4 ± 4.7	56.9 ± 4.4
*P* values	<0.001*	<0.001*	<0.001*	<0.001*
Post hoc *P* values	1-2; <0.001*	1-2; <0.001*	1-2; <0.001*	1-2; <0.001*
	1-3; <0.001*	1-3; <0.001*	1-3; <0.001*	1-3; <0.001*
	1-4; <0.001*	1-4; <0.001*	1-4; <0.001*	1-4; <0.001*

* *P* < 0.001 Its difference from the control group is significant.

**Figure 5:**
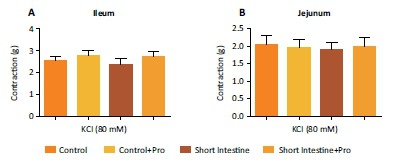
The changes induced by Krebs bicarbonate solution (vehicle), L-NNA, indomethacin, nimesulide, hexamethonium, and tetrodotoxin in the frequency values of spontaneous ileal smooth muscle contractions obtained in rats from all groups. A - Control, B - Short intestine, C - Control pro, D - Short intestine pro. The findings were presented as the mean ± standard deviation of 8 experiments. * *P* <0.001 Its difference from the control group is significant.

**Figure 6:**
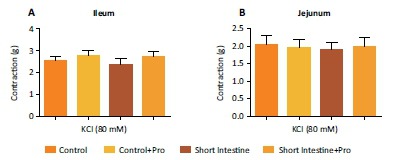
The changes induced by Krebs bicarbonate solution, L-NNA, indomethacin, nimesulide, hexamethonium, and tetrodotoxin in the frequency values of spontaneous jejunal smooth muscle contractions obtained from rats in all groups. A - Control, B - Short intestine, C - Control pro, D - Short intestine pro. The findings were presented as the mean ± standard deviation of 8 experiments. * *P* < 0.001 Its difference from the control group is statistically significant.

We observed that the frequency responses returned to normal in the ileum and jejunum treated with probiotics. A significant difference was detected across the groups after antagonist medications were added (For the ileum; *P* <0.001, *P* <0.001, *P* <0.001, *P* <0.001, respectively), (For the jejunum; *P* <0.001, *P* <0.001, *P* <0.001, *P* <0.001, respectively). While L-NNA increased frequency responses in the ileum; indomethacin, nimesulide, hexamethonium, and tetrodotoxin made no significant difference. Whereas in the jejunum, L-NNA, indomethacin, and nimesulide increased frequency responses but hexamethonium and tetrodotoxin made no change (Tables 6 and 7) (Figs. 5 and 6).

**Table 6:** Frequency changes in the ileal tissue in the presence of antagonists (number/10 minutes). The changes induced by Krebs bicarbonate solution (vehicle), L-NNA, indomethacin, nimesulide, hexamethonium, and tetrodotoxin in the frequency values of ileal spontaneous smooth muscle contractions obtained in all groups are shown in Group 1, Group 2, Group 3, and Group 4. Before the measurement, all tissues were incubated with antagonist agents for 30 minutes. The findings were presented as the mean ± standard deviation of 8 subjects.

Ileum	Control	Short intestine	Pro + control	Pro + Short intestine
Vehicle	27.2 ± 4.2	42.8 ± 3.9	24.0 ± 3.6	32.6 ± 4.1
L-NNA	44.8 ± 4.5*	56.6 ± 4.0*	37.2 ± 3.5*	47.2 ± 4.0*
Indomethacin	32.2 ± 4.0	45.4 ± 3.8	27.2 ± 3.2	36.8 ± 4.0
Nimesulide	30.7 ± 4.1	44.4 ± 3.6	25.9 ± 3.3	34.2 ± 3.9
Hexamethonium	24.4 ± 3.8	36.8 ± 4.5	20.6 ± 3.5	29.0 ± 4.1
Tetrodotoxin	25.0 ± 4.0	35.7 ± 4.0	18.8 ± 4.0	27.0 ± 4.2
*P* values	<0.001*	<0.001*	<0.001*	<0.001*
Post hoc *P* values	1-2; <0.001*	1-2; <0.001*	1-2; <0.001*	1-2; <0.001*

* *P* <0.001 Its difference from the control group is significant.

**Table 7:** The frequency changes in the presence of antagonists in jejunal tissue (number/10 minute). The changes induced by Krebs bicarbonate solution (vehicle), L-NNA, indomethacin, nimesulide, hexamethonium, and tetrodotoxin in the frequency values of spontaneous jejunal smooth muscle contractions obtained in rats from all groups are shown in Group 1, Group 2, Group 3, and Group 4. Before the measurement, all tissues were incubated with antagonist agents for 30 minutes. The findings were presented as the mean ± standard deviation of 8 subjects.

Jejunum	Control	Short intestine	Pro + control	Pro + Short intestine
Vehicle	20.2 ± 3.0	29.2 ± 3.2	17.8 ± 3,4	23.8 ± 3.3
L-NNA	30.6 ± 3.2*	44.5 ± 4.0*	28.0 ± 3.0*	37.2 ± 3.4*
Indomethacin	27.2 ± 3.0*	40.0 ± 4.0*	25.2 ± 2.9*	34.0 ± 3.0*
Nimesulide	26.8 ± 3.2*	38.2 ± 3.9*	24.4 ± 3.1*	32.2 ± 2.8*
Hexamethonium	17.3 ± 2.8	26.0 ± 3.1	15.0 ± 2.8	19.8 ± 3.0
Tetrodotoxin	15.4 ± 3.0	24.2 ± 3.7	14.1 ± 2.6	18.8 ± 2.9
*P* values	<0.001*	<0.001*	<0.001*	<0.001*
Post hoc *P* values	1-2; <0.001*	1-2; <0.001*	1-2; <0.001*	1-2; <0.001*
	1-3; <0.001*	1-3; <0.001*	1-3; <0.001*	1-3; <0.001*
	1-4; 0.003*	1-4; <0.001*	1-4; 0.003*	1-4; <0.001*

* *P* < 0.01 Its difference from the control group is significant.

## Discussion

In our study, we observed that the short bowel contraction responses increased in the experimental SBS model and that the increased contraction responses returned to normal levels after administering probiotics. We examined the mechanisms that changed the contraction responses by adding L-NNA, indomethacin, nimesulide, hexamethonium, and tetrodotoxin to the medium. We believe that probiotics modify the contraction responses of short bowels by means of NO and COX products.

In the first phase of our study, the KCl contraction responses of all ileal and jejunal tissue samples obtained from the groups were evaluated. No significant difference was detected between the responses. The fact that the smooth muscle structures of all the samples obtained responded similarly to KCl indicated tissue integrity and that the KCl contraction mechanism that operates through the Ca+2 channels was intact. With a clearer expression, it was shown that the experimental model induced did not disrupt physiological contraction mechanisms. The increased bowel transit time that develops in relation to SBS is an increase in motility not only because the length of the bowel has shortened but also because amplitude and frequency have increased. Besides this, it was observed that amplitude and frequency reverted to the control group values in the short bowel + probiotic group. This observation suggests that feeding with probiotics in clinical practice reduces the bowel transit time by reducing the smooth muscle amplitude and frequency. While in the ileal tissue L-NNA increased amplitude values significantly in each of the groups when compared to the control group, the frequency values were increased by L-NNA, indomethacin, and nimesulide. In the jejunal tissue, L-NNA, indomethacin, and nimesulide increased amplitude and frequency. In the jejunum and ileum, hexamethonium and tetrodotoxin did not change the amplitude and frequency values. The products of the nitrergic system and COX control the amplitude and frequency inhibition of spontaneous contractions.

NO plays a major role in many physiological and pathological events in the gastrointestinal system (GIS) ^30^ . NO released from the neurons of the myenteric plexus in the GIS relaxes intestinal smooth muscles. This effect is particularly significant in the pylorus, gastroesophageal sphincter, and the Sphincter of Oddi ^31 - 33^ . L-NNA is a chemical substance that inhibits the nitric oxide synthase (NOS) enzyme that participates in the formation of NO. In our study, L-NNA increased the amplitude contractions in the ileum and the jejunum of each group. This result suggests that NO has a reductive effect on amplitude formation in the ileal and jejunal tissues. Statistically, L-NNA has increased amplitude responses in the short bowel groups less that it did in control groups; therefore, the increase in amplitudes in cases of short bowel may be associated with decreased NO. The delivery of probiotics has reduced the increased amplitude to its normal values in both tissues. This suggests that the administration of probiotics in short bowels brings the increased amplitude levels closer to the control levels by increasing the synthesis of NO.

Previous studies report that COX pathways play a role in the regulation of GIS motility ^34^ and that COX-2 may also contribute to the pathophysiology of motor changes ^35 - 37^ . For example, the creation of experimental ileus stimulated by bowel manipulation was related to COX-2 induction, and treatment with selective COX-2 inhibitors regulate intestinal contractility both in vivo and in vitro ^35^ . Indomethacin is a medication that inhibits the enzymes cyclooxygenase (COX) 1 and 2, whereas nimesulide inhibits the synthesis of prostaglandins only by inhibiting the COX-2 enzyme. In our study, the amplitude contractions were increased by indomethacin and nimesulide in the ileal and jejunal preparations of each group. This result shows that the increase of prostaglandins reduces amplitude contractions in both tissues. This is because the medications that reduce the production of prostaglandins have increased the amplitude contractions significantly in comparison to control values. It appears that the increase of prostaglandins is particularly related to the COX-2 enzyme. The only case where no statistically significant difference was detected regarding increased amplitude contractions was between nimesulide that only inhibits the COX-2 enzyme and indomethacin that inhibits COX-1 and COX-2 enzymes.

Hexamethonium is a medication that blocks nicotinic receptors present in ganglions. In our study, hexamethonium did not alter the amplitude and frequency contractions in the ileal and jejunal preparations in each of the groups. This result shows that the stimulation of nicotinic receptors has no effect on the generation of amplitude and frequency.

Tetrodotoxin is a chemical substance that prevents the generation of an action potential by manifesting an effect similar to that of local anesthetic medications by blocking voltage-gated sodium channels. In our study, tetrodotoxin did not alter the amplitude and frequency contractions in the ileal and jejunal preparations of each group. This result implies that the generation of amplitude and frequency in both tissues is not an effect related to the generation of action potential.

Microorganisms used as probiotics are mostly from the Lactobacillus and bifidobacteria group that are essential components of the human gastrointestinal flora. However, some nonpathogenic streptococci, *Escherichia coli* , some strains of *Enterococcus faecium* , and some yeasts such as *Saccharomyces boulardi* have been used in the preparation of probiotic preparations ^38^ . Probiotics can be defined as supportive live microorganisms that stimulate the immune system by regulating intestinal flora and benefit health. This beneficial effect of probiotics has been associated with the fact that they create a mucosal barrier against enteropathogens ^39 - 51^ , immunostimulation and modulation ^8 - 13^ , their anticarcinogenic and antimutagenic activity, their improvement of lactose use, and the fact that they reduce cholesterol ^52^ . Clinical and experimental studies conducted have shown that probiotics can be used in infective diarrhea ^53 - 56^ , antibiotic diarrhea ^57 - 59^ , travel diarrhea ^60 - 62^ , irritable bowel syndrome ^63 - 67^ , and inflammatory bowel diseases ^68 - 72^ . In a study conducted on rats, it was shown that the addition of probiotics or probiotics to the diet influences myoelectrical activity and changes the bacterial composition ^73^ . Most of the beneficial effects of probiotics identified are based on clinical research. The effects of probiotic extracts on intestinal tissues placed in organ baths have been researched by Massi *et al* . ^29^ . In this study, it was identified that only Lactobacillus increased ileal contractions and that Lactobacillus, Bifidobacterium, and Streptococcus strains reduce proximal colon contractions. It has been shown that probiotic bacteria perform this effect via cytoplasmic fraction. Our study took one more step ahead of that study and researched the effects of the three-week use of probiotics on intestines. With consideration of other studies as well, the benefits we identified are caused by the effects of probiotics on the intestinal wall.

## Conclusions

As a result, in the early three-week period after surgically creating a short bowel, motility is increased by increasing amplitude and frequency in both the ileum and the jejunum. The administration of probiotics in this early period contributes to reducing the motility that has increased. By researching the changes in intestinal motility and its reasons in the following adaptation periods of SBS, more benefits can be provided for the treatment of this disease. Besides this, if motility studies are conducted at the level of cellular receptors, the results will be much more beneficial for explaining SBS.

## Footnotes

1 Research performed at Department of Pediatric Surgery, Faculty of Medicine, Cumhuriyet University, Sivas, Turkey.
